# Reduced bone morphogenic protein signaling along the gut–neuron axis by heat shock factor promotes longevity

**DOI:** 10.1111/acel.13693

**Published:** 2022-08-17

**Authors:** Sonja L. B. Arneaud, Jacob McClendon, Lexus Tatge, Abigail Watterson, Kielen R. Zuurbier, Bhoomi Madhu, Tina L. Gumienny, Peter M. Douglas

**Affiliations:** ^1^ Department of Molecular Biology UT Southwestern Medical Center Dallas Texas USA; ^2^ Department of Biology Texas Woman's University Denton Texas USA; ^3^ Hamon Center for Regenerative Science and Medicine UT Southwestern Medical Center Dallas Texas USA

**Keywords:** aging, BMP signaling, endocytosis, gut–neuron axis, HSF‐1, membrane traffic, Rab GTPases, SMAD, TGF‐β

## Abstract

Aging is a complex and highly regulated process of interwoven signaling mechanisms. As an ancient transcriptional regulator of thermal adaptation and protein homeostasis, the Heat Shock Factor, HSF‐1, has evolved functions within the nervous system to control age progression; however, the molecular details and signaling dynamics by which HSF‐1 modulates age across tissues remain unclear. Herein, we report a nonautonomous mode of age regulation by HSF‐1 in the *Caenorhabditis elegans* nervous system that works through the bone morphogenic protein, BMP, signaling pathway to modulate membrane trafficking in peripheral tissues. In particular, HSF‐1 represses the expression of the neuron‐specific BMP ligand, DBL‐1, and initiates a complementary negative feedback loop within the intestine. By reducing receipt of DBL‐1 in the periphery, the SMAD transcriptional coactivator, SMA‐3, represses the expression of critical membrane trafficking regulators including Rab GTPases involved in early (RAB‐5), late (RAB‐7), and recycling (RAB‐11.1) endosomal dynamics and the BMP receptor binding protein, SMA‐10. This reduces cell surface residency and steady‐state levels of the type I BMP receptor, SMA‐6, in the intestine and further dampens signal transmission to the periphery. Thus, the ability of HSF‐1 to coordinate BMP signaling along the gut–brain axis is an important determinate in age progression.

## Graphical abstract

Reduced DBL‐1/BMP signaling by neural hsf‐1 promotes a negative feedback loop in the intestine by reducing cell surface residency of the dedicated DBL‐1 receptor, SMA‐6, via the repression of membrane trafficking machinery.

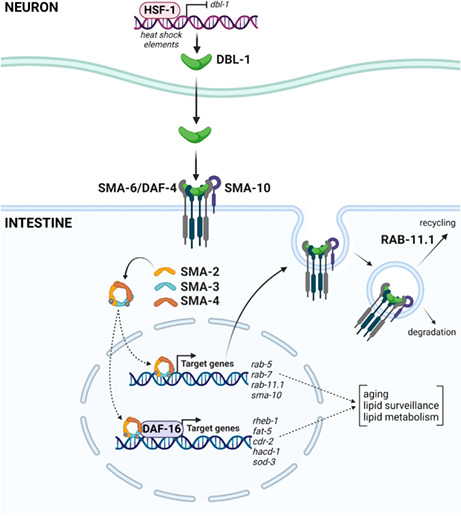

**Abbreviations**
BMP: Bone morphogenic protein
EV: Empty vector
FUdR: 5‐fluorouracil‐2'‐deoxyribose
GFP: Green fluorescent protein
HSF: Heat shock factor
LRIG: Leucine rich and immunoglobulin domains
NGM: Nematode growth medium
qPCR: Quantitative reverse‐transcriptase PCR
TB: Terrific broth
TGF: Transforming growth factor
WT: wild‐type

## 1 INTRODUCTION

Rates of tissue aging within an animal are coordinated through a complex network of intra‐ and intercellular signaling pathways. Due to the complexity of this multifactorial aging mechanism, a variety of molecular targets have been identified as age determinates across a number of model organisms ranging from yeast to primates (Kenyon, 2010). In multicellular organisms, the ability of these molecules to act across tissues and connect organ systems plays a critical role in animal physiology and age determination. The nervous system has long been appreciated as the hub of information transfer and communication between the body's other organ systems (Miller et al., 2020). Modulating critical regulators of cellular adaption to stress in the nervous system initiates systemic alterations with cytoprotective and age‐defying potential (Douglas et al., 2015; Durieux et al., 2011; Taylor & Dillin, 2013). Receipt of these neural‐born signals in the peripheral tissues is essential for the transmission and initiation of distinct cellular responses. Communication between the brain and intestine has become increasingly appreciated in the context of human health and can occur through direct synaptic innervation of the tissues, neuroendocrine means, systemic fluctuations in metabolism, or the immune response (Ambrosini et al., 2019; Boehme et al., 2020; Carabotti et al., 2015; Jena et al., 2020). To date, the molecular and cellular details underlying these integrated, trans‐organ signaling mechanisms and their role in age regulation remain unclear.

The heat shock transcription factor, HSF‐1, is an ancient regulator of cellular adaption to thermal stress with evolved roles as an aging regulator in the nervous system of the nematode, *Caenorhabditis elegans* (Morimoto, 1998; Morley & Morimoto, 2004). While HSF‐1 activates the expression of protein folding enzymes to ensure proteome integrity amid thermal destabilization (Akerfelt et al., 2010; Morley & Morimoto, 2004), it has also developed as an important aging regulator in part through its ability to cooperate with the FOXO transcription factor, DAF‐16 (Hsu et al., 2003). Elevating the expression of HSF‐1 exclusively within the nervous system extends animal lifespan and requires the activity of enteric DAF‐16 (Douglas et al., 2015; Morley & Morimoto, 2004). Neural *hsf‐1* was recently shown to reduce signaling through the bone morphogenic protein (BMP) pathway for adaptation to elevated, nonpermissive temperatures (Chauve et al., 2021). As a member of the greater transforming growth factor β (TGF‐β) signaling pathway in *C. elegans*, DBL‐1 is a neural‐produced BMP ligand (Morita et al., 1999; Suzuki et al., 1999) that signals to peripheral tissues through its binding to the heterodimeric receptor complex composed of the type 1 receptor, SMA‐6, and type 2 receptor, DAF‐4 (Gumienny & Savage‐Dunn, 2013; Roberts et al., 2010; Wang et al., 2002). In addition to regulating body size and mail tail development (Morita et al., 1999; Suzuki et al., 1999), DBL‐1 has been linked with olfactory learning, reproductive aging, lipid metabolism, and the innate immune response (Clark et al., 2018; Luo et al., 2010; Zhang & Zhang, 2012; Zugasti & Ewbank, 2009). DBL‐1 signal transduction in peripheral tissues acts through the nucleocytoplasmic SMAD proteins (SMA‐2, SMA‐3, and SMA‐4)(Gumienny & Savage‐Dunn, 2013). SMA‐3, in particular, co‐occupies some of the same DNA regulator elements as DAF‐16 and jointly regulates the expression of select genes (Qi et al., 2017). Since DAF‐16 is required for many DBL‐1 functions including larval development (Kaplan et al., 2015), response to environmental nanopolystyrene (Liu et al., 2020), and lipid accumulation (Clark et al., 2018, 2021; Liu et al., 2020), we sought to determine whether DBL‐1 signaling might play an important role in HSF‐mediated age regulation.

**FIGURE 1:**
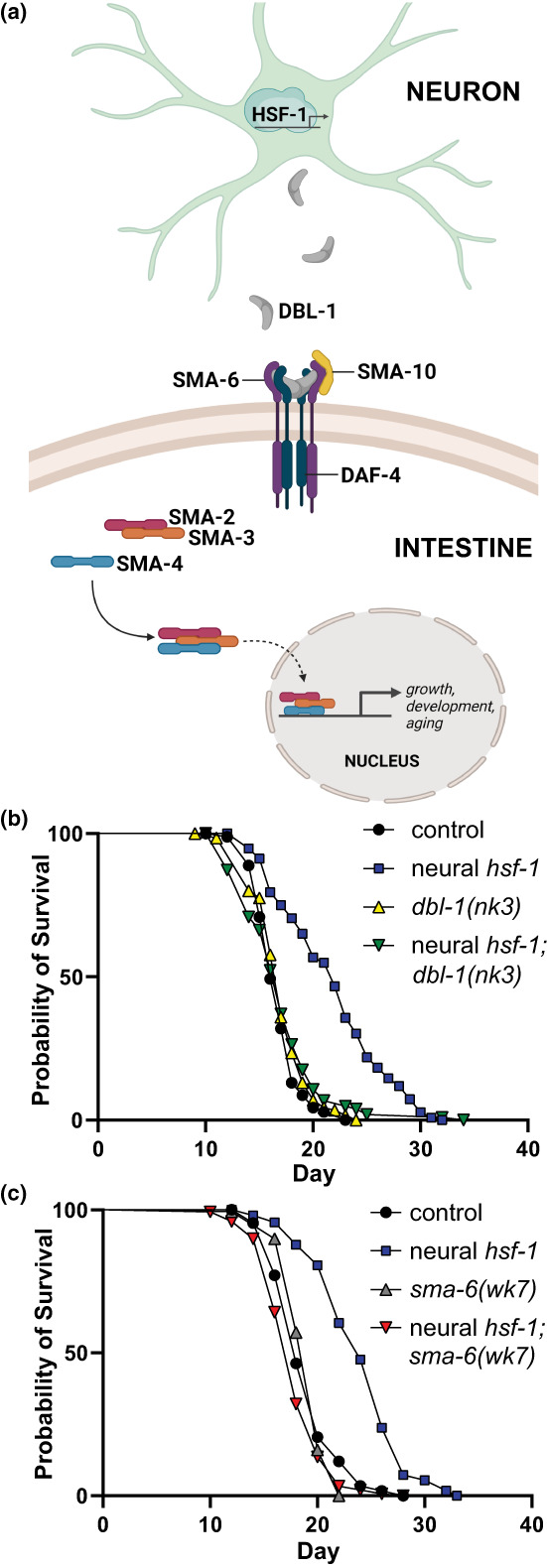
BMP signaling across tissues is required for lifespan extension by neural *hsf‐1*. (a) Schematic representing the BMP/DBL‐1 signaling pathway in *Caenorhabditis elegans*. (b,c) Lifespan analysis of neural *hsf‐1* transgenic worms at 20°C in either the *dbl‐1(nk3)*, b, or *sma‐6(wk7)*, c, mutant background. See Table S1.

Herein we report that the nonautonomous mode of age regulation by neural HSF‐1 acts through this BMP/DBL‐1 signaling pathway (Figure 1a). Both the neuron‐specific ligand, DBL‐1, and its dedicated receptor in the intestine, SMA‐6, were required for lifespan extension by neural HSF‐1. We find that neural HSF‐1 represses *dbl‐1* transcription thereby reducing BMP signal transduction in the intestine via a SMA‐3‐mediated negative feedback loop entailing the transcriptional repression of membrane trafficking regulators linked to intracellular lipid surveillance (Watterson et al., 2022). In turn, this reduces steady‐state protein levels of the SMA‐6 receptor and further dampens signal transduction. Our studies are consistent with another report that impaired DBL‐1 signaling extends *C. elegans* lifespan (Luo et al., 2009). Moreover, the ability of HSF‐1 to coordinate BMP/DBL‐1 signaling across tissues resides upstream of enteric DAF‐16. While previous reports have demonstrated that neural *hsf‐1* utilizes a distinct transcellular signaling mechanism involving chaperone activation to protect against acute heat stress (Douglas et al., 2015), thermal adaption by neural *hsf‐1* under mild yet chronic heat stress conditions at 25°C appears to utilize a similar DBL‐1 dependent mechanism (Chauve et al., 2021). Consistent with these previous studies, we identify the neuron‐specific BMP ligand, DBL‐1, as a critical signaling molecule required for neural HSF‐1 to extend lifespan and report how its transmission to the intestine modulates surface residency of the SMA‐6 receptor through a SMAD negative feedback loop.

## 2 RESULTS

### 2.1 Neural *hsf‐1* requires both the DBL‐1 ligand and its intestinal SMA‐6 receptor for lifespan extension

Mutations of *dbl‐1* and its dedicated type I transmembrane serine/threonine kinase receptor, SMA‐6, are involved in age modulation (Luo et al., 2009). Moreover, neural *hsf‐1* was recently shown to reduce DBL‐1 signaling at the nonpermissive temperature of 25°C (Chauve et al., 2021). We hypothesized that this particular BMP signaling mechanism is required for lifespan extension by neural *hsf‐1*. Indeed, the *dbl‐1(nk3)* null mutation abolished lifespan extension by neural *hsf‐1* (Figure 1a,b, Figure S1a, Table S1). While *dbl‐1* expression was necessary for lifespan extension by neural *hsf‐1*, we next examined its sufficiency. Under our experimental conditions, elevating the expression or activity of *dbl‐1* through transgenic *dbl‐1::GFP* overexpression (Schultz et al., 2014) or RNAi of its negative regulator, *lon‐2* (Gumienny et al., 2007; Taneja‐Bageshwar & Gumienny, 2013) did not impact lifespan and does not appear to be sufficient to promote lifespan extension (Figure S1b–d and Table S1). Due to the requirement of this BMP signaling ligand to extend lifespan, we next examined the necessity of its dedicated type I receptor, SMA‐6, in the peripheral tissues (Figure 1a). Consistent with DBL‐1 being required for lifespan extension, preventing receipt of this neural‐born DBL‐1 ligand via *sma‐6(wk7)* mutation also abolished lifespan extension by neural *hsf‐1* (Figure 1c, Figure S1e, and Table S1). Previous studies have highlighted the importance of the intestine for neural *hsf‐1* to extend animal lifespan (Douglas et al., 2015). Rescuing *sma‐6::GFP* expression exclusively in the intestine was sufficient for the neural *hsf‐1* to extend the lifespan in the *sma‐6(wk7)* mutant background (Figure S1f and Table S1). Thus, neural *hsf‐1* requires both the neural‐born DBL‐1 ligand and its dedicated BMP receptor, SMA‐6, in the intestine to modulate the aging process. Yet how neural *hsf‐1* modulates this transcellular signaling paradigm was unclear.

### 2.2 Neural HSF‐1 represses expression of the BMP ligand, DBL‐1

**FIGURE 2:**
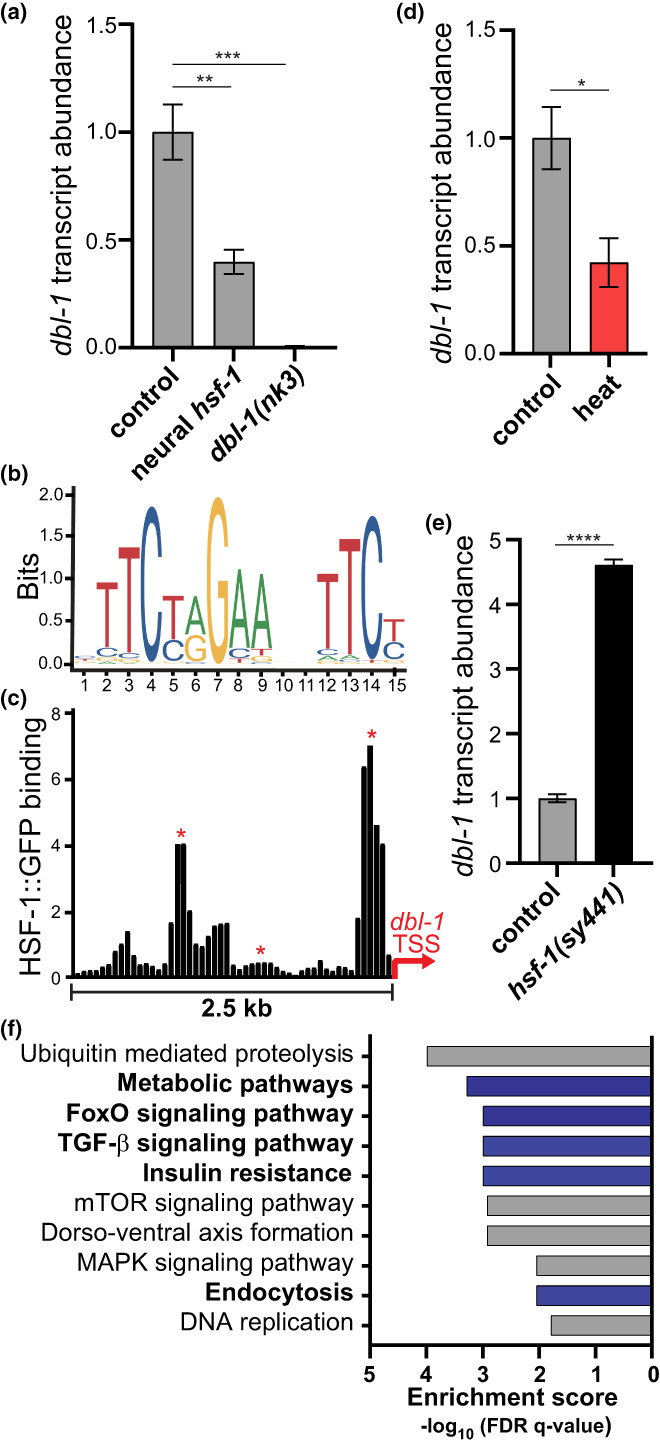
HSF‐1 represses transcription of the neural‐born BMP ligand, DBL‐1. (a) Relative *dbl‐1* transcript abundance in the respective mutant and transgenic strains determined by RNAseq. Mean ± SEM, ***p = 0.0003 and ** *p* = 0.0034 by one‐way ANOVA with the Dunnett's multiple comparison test. *n* = 3. (b) Schematic of predicted consensus heat shock element in the 5′ UTR of *dbl‐1*. (c) Enrichment of HSF‐1::GFP binding upstream of the DBL‐1 transcriptional start sites (TSS) (2.5 kb) determined by previously reported HSF‐1::GFP chromatin immunoprecipitation. Red asterisks highlight predicted Heat Shock Elements (HSE). (d, e) Relative *dbl‐1* transcript abundance after transient heat shock determined by RNAseq, d, and RT‐qPCR, e. Mean ± SEM, *p = 0.0391 by two‐way ANOVA with the Sidak multiple comparison test (reference Figure S2c) and ****p ˂ 0.0001 by the unpaired *t*‐test. *n* = 2, d, *n* = 3, e. (f) KEGG pathway analysis from DAVID Bioinformatic's Resource v6.8. Plot displays the top 10 over‐represented terms from genes significantly regulated in neural *hsf‐1* transgenic animals (*p* ˂ 0.05).

As a well‐established regulator of worm body length, DBL‐1 expression levels directly correlate with animal body length (Morita et al., 1999; Suzuki et al., 1999; Wang et al., 2002). Transgenic animals expressing *hsf‐1* in the nervous system demonstrated smaller body size throughout development and a 10% reduction in body length as egg‐laying Day 1 adults (Figure S2a,b). Since neural *hsf‐1* was reported to modulate DBL‐1 activity (Chauve et al., 2021), we examined whether these morphological differences in body size correspond with *dbl‐1* expression levels. Consistent with this prior study (Chauve et al., 2021), analysis of transcriptomic datasets confirmed a 2‐fold reduction in *dbl‐1* expression in neural *hsf‐1* transgenic animals (Figure 2a). By contrast, transcript levels for a different TGF‐β signaling ligand, DAF‐7, were not changed in neural *hsf‐1* transgenic animals, further highlighting the selectivity of HSF‐1 in the BMP branch of the greater TGF‐β signaling pathway (Figure S2c). Three predicted consensus heat shock elements were identified within the 5′ untranslated region of DBL‐1, suggesting a direct mode of transcriptional regulation (Figure 2b). Analysis of previously reported ChIP‐seq datasets (Labbadia & Morimoto, 2015) revealed enrichment of HSF‐1 binding at two of the three predicted heat shock elements within the DBL‐1 promoter (Figure 2c). In a complementary fashion, activation of HSF‐1 via transient heat shock reduced *dbl‐1* transcript abundance but had no significant effect on *daf‐7* transcription (Figure 2d and Figure S2d). Conversely compromising *hsf‐1* activity via the *hsf‐1(sy441)* hypomorphic mutation activated *dbl‐1* transcription by over 4‐fold (Figure 2e). Comparison of transcriptional profiles between *dbl‐1(nk3)* null mutants and neural *hsf‐1* transgenic animals showed a significant overlap of differentially regulated genes (Figure S2e–g and Table S2). Moreover, analysis of transcripts differentially regulated in neural *hsf‐1* animals identified TGF‐β signaling (cel04350) as the 4th most significantly enriched KEGG pathway (Figure 2f). Consistent with HSF‐1 and its dependence on the DAF‐16/FOXO transcription factor for age regulation (Douglas et al., 2015; Hsu et al., 2003), both FOXO signaling (cel04068) and insulin resistance (cel04931) were significantly enriched in KEGG pathway analysis (Figure 2f). Collectively, these data suggest that neural *hsf‐1* binds the DBL‐1 promoter and mediates its transcriptional repression resulting in physiological defects regarding body length.

### 2.3 Neural *hsf‐1* reduces peripheral DBL‐1 signaling

**FIGURE 3:**
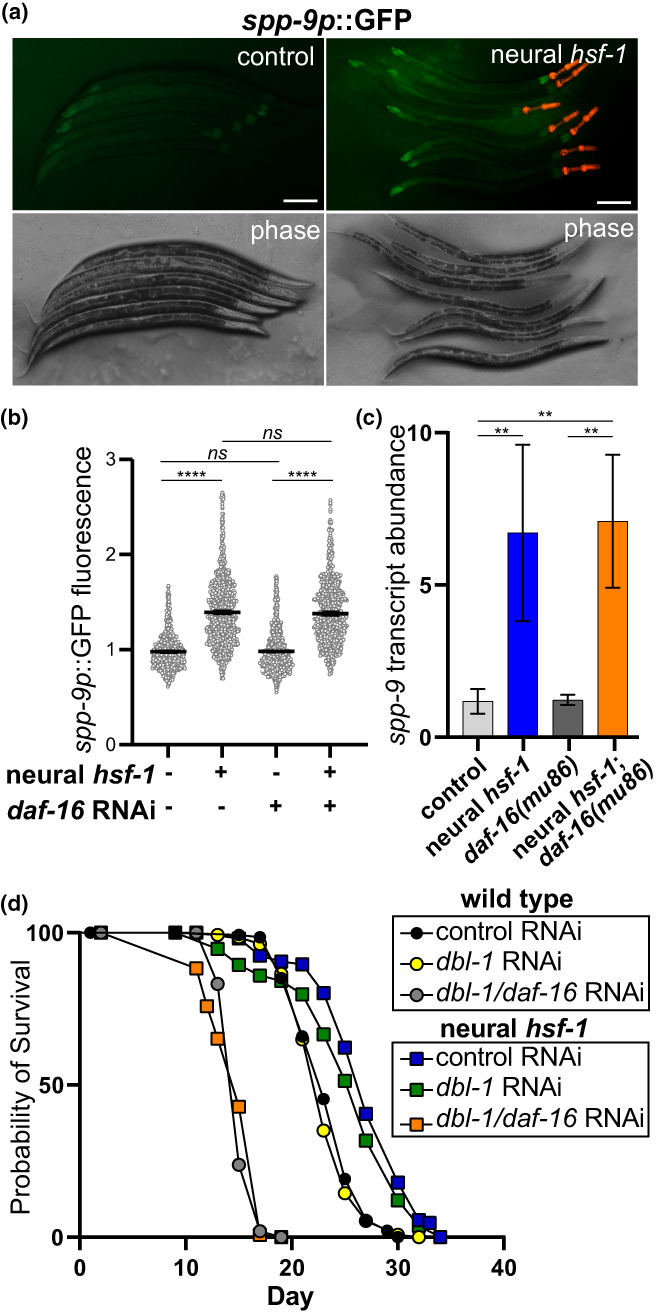
Neural *hsf‐1* represses DBL‐1 signaling in the peripheral tissues independent of DAF‐16. (a, b) Transgenic Day 1 adult worms harboring the *spp‐9p*::GFP transcriptional reporter in wild‐type, WT, (PMD145) and neural *hsf‐1* (PMD93) backgrounds. (a) Fluorescence micrographs, scale = 100 μm and (b) Relative *spp‐9p*::GFP fluorescence by large‐particle flow cytometry. Worms cultured on control empty vector or *daf‐16* RNAi. Mean with 95% confidence interval, ****p ˂ 0.0001 and *ns*, not significant by one‐way ANOVA with the Tukey test, *n* = 924 (PMD144, EV), *n* = 1843 (PMD144, *daf‐16* RNAi), *n* = 1282 (PMD64, EV) and *n* = 993 (PMD64, *daf‐16* RNAi) over multiple independent trials. (c) Relative transcript abundance for s*pp‐9* determined by qPCR. Wild type or control (light gray, N2), neural *hsf‐1* (blue), *daf‐16(mu86)* (dark gray), and *daf‐16(mu86)*; neural *hsf‐1* (orange). Left to right, **p = 0.0074, 0.0039, and 0.0043 by two‐way ANOVA with the Sidak multiple comparison test, *n* = 3. (d) Lifespan analysis of control (N2) and neural *hsf‐1* transgenic worms at 20°C with the respective RNAi combinations. See Table S1.

Since HSF‐1 represses expression of the neural‐produced DBL‐1 ligand, we examined how these transcriptional changes in the nervous system elicited a complementary response in the animal's peripheral tissues (Wang et al., 2002). Worm morphology suggested that neural *hsf‐1* initiated a periphery response as indicated by shortened body length of adults (Figure S2a,b). Monitoring whole‐body fluorescence from two distinct DBL‐1 pathway transcriptional reporters, *spp‐9p*::GFP (Roberts et al., 2010) and *RAD/SMADp*::GFP::NLS (Tian et al., 2010), enabled the spatial characterization and quantification of DBL‐1 signaling to peripheral tissues. In keeping with *spp‐9* expression being inversely proportional to DBL‐1 levels (Madhu et al., 2020; Roberts et al., 2010), we observed enhanced *spp‐9p*::GFP fluorescence in transgenic animals expressing neural *hsf‐1* (Figure 3a,b and Figure S3a), indicating a reduction in DBL‐1 signaling. We also observed that expression of full‐length *hsf‐1* in the nervous system was sufficient to activate *spp‐9* transcription in the *hsf‐1(sy441)* hypomorphic background (Figure S3b), further supporting the neuronal origins of the DBL‐1 signaling mechanism. Consistent with previous reports (Chauve et al., 2021), neural *hsf‐1* transgenic animals showed reduced fluorescence from the RAD/SMAD transcriptional reporter (Tian et al., 2010) throughout larval development and into day 1 of adulthood (Figure S3c–e), further confirming that neural *hsf‐1* reduces DBL‐1/BMP signaling in peripheral tissues.

We then asked whether DAF‐16/FOXO, which is required for lifespan extension by neural *hsf‐1* (Douglas et al., 2015), is also needed for DBL‐1 signaling. Reduced expression of *daf‐16* via RNAi treatments did not impact *spp‐9p*::GFP fluorescence in the animal's peripheral tissues (Figure 3b), suggesting it may act downstream of, or in parallel with, DBL‐1 signaling to impact age regulation. In further support, activation of *spp‐9* transcription by neural *hsf‐1* still occurred in *daf‐16(mu86)* null mutant animals (Figure 3c). To examine the combined impact of FOXO and BMP signaling on lifespan, co‐administering *daf‐16* and *dbl‐1* RNAi significantly reduced lifespan when compared to *dbl‐1* alone (Figure 3d and Table S1), highlighting that both BMP and FOXO signaling might serve distinct yet complementary roles with respect to lifespan determination by neural *hsf‐1*. Overall, repression of *dbl‐1* transcription by neural *hsf‐1* reduces systemic BMP signaling seemingly independent of DAF‐16.

### 2.4 Intestinal SMA‐6 protein levels are reduced through repressed membrane trafficking

Canonical DBL‐1/BMP signal transduction is composed of multiple transmembrane serine/threonine kinase receptors. Upon ligand binding, the type I BMP receptor, SMA‐6, and its complementary type II transmembrane receptor, DAF‐4, are recruited into a heterotetrameric complex at the plasma membrane which in turn activates the intracellular receptor‐regulated r‐SMAD proteins, SMA‐2 and SMA‐3. SMA‐2, SMA‐3, and co‐Smad SMA‐4 then form a complex that enters the nucleus and regulates transcription of target genes (Gumienny & Savage‐Dunn, 2013) (Figure 1a). While *daf‐4* transcript levels remained unchanged, we observed DBL‐1‐dependent transcriptional activation of *sma‐6* in neural *hsf‐1* transgenic worms (Figure S4a). Conversely, the DAF‐7 type I receptor, *daf‐1*, which also heterodimerizes with DAF‐4, is repressed in a DBL‐1‐dependent manner (Figure S4a), suggesting a potential inverse relationship between signaling by DBL‐1 versus DAF‐7. However, further studies are required to elucidate the interplay between these different TGF‐β signaling pathways.

**FIGURE 4:**
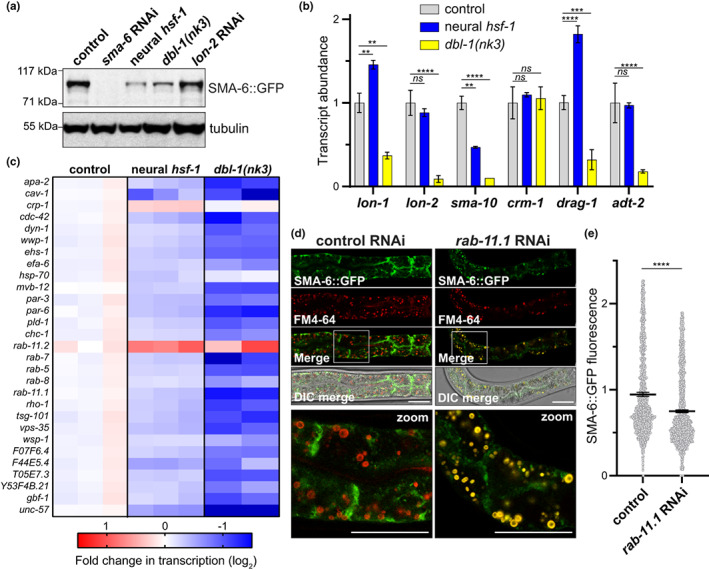
Reduced DBL‐1 signaling by neural *hsf‐1* decreases steady‐state SMA‐6 levels through increased receptor internalization. (a) Western blot analysis of L4 larval transgenic worms expressing intestinal SMA‐6::GFP in either control (RT2495), neural *hsf‐1* (PMD35), or *dbl‐1(nk3)* mutant (PMD87) backgrounds. Transgenic worms were treated with *sma‐6* or *lon‐2* RNAi. (b) Relative transcript abundance of cell surface and extracellular regulators of BMP signaling in wild‐type (gray, N2), neural *hsf‐1* (blue, AGD1289), and *dbl‐1(nk3*) mutant (yellow, NU3) animals. Mean ± SEM. From left to right, **p = 0.0096, **p = 0.0015, **p = 0.0028, ***p = 0.0007, ****p ˂ 0.0001 and *ns* = not significant with *p* ˃ 0.05 by two‐way ANOVA with the Dunnett's multiple comparison test, *n* = 3 (wild‐type and neural *hsf‐1*) and *n* = 2 for *dbl‐1(nk3)* mutants. (c) Heat map depicts relative fold change in transcript abundance in wild‐type control (N2), neural *hsf‐1* transgenic (AGD1289), and *dbl‐1(nk3)* mutant (NU3) worms. Shown are annotated endocytosis‐related transcripts (cel04144) differentially regulated in neural *hsf‐1* transgenic animals (*p* ˂ 0.05). Red = activation, blue = repression. (d) Fluorescence micrographs of Day 1 adult transgenic worms expressing intestinal SMA‐6::GFP (PMD28) on empty vector control or *rab‐11.1* RNAi. Endocytic vesicles are marked with the FM4‐64 dye (red). Scale = 25 μm. (e) Relative fluorescence in Day 1 adult transgenic worms expressing SMA‐6::GFP by large‐particle flow cytometry. Mean with 95% confidence interval, ****p ˂ 0.0001 by an unpaired student's *t*‐test, *n* = 1605 (control) and *n* = 2540 (*rab‐11.1* RNAi) over multiple trials

Despite elevated *sma‐6* transcript abundance upon neural *hsf‐1* expression, we observed that steady‐state protein levels of ectopically expressed SMA‐6::GFP in the intestine were reduced by 79% at late L4 larval/Day 1 adult stages as indicated by western blot analysis (Figure 4a and Figure S4b). Consistent with reducing *dbl‐1* signaling by neural *hsf‐1*, *dbl‐1(nk3)* mutants showed a similar 72% decrease in enteric SMA‐6::GFP protein levels, while pathway activation via *lon‐2* RNAi did not significantly affect SMA‐6::GFP levels (Figure 4a and Figure S4b). We confirmed that reducing *sma‐6* expression via RNAi was sufficient to decrease BMP signal transduction in peripheral tissues as evidenced by increased fluorescence of the *spp‐9p*::GFP reporter strain (Figure S4c). Thus, reducing *dbl‐1* expression by neural *hsf‐1* or gene deletion compromises steady‐state protein levels of its dedicated SMA‐6 receptor, which corresponds with reduced BMP signaling.

Intrigued by the loss in intestinal SMA‐6::GFP protein levels by neural *hsf‐1*, we further investigated how reducing BMP signaling by neural *hsf‐1* might impact steady‐state protein levels of the SMA‐6 receptor. Several regulators of BMP/TGF‐β signaling have the capacity to bind and modulate BMP/DBL‐1 receptor dynamics, including trafficking to endosomes for recycling or degradation (Gumienny & Savage‐Dunn, 2013). Thus, we examined whether reduced DBL‐1 signaling by neural *hsf‐1* and signal ablation in *dbl‐1(nk3)* mutants elicited a dose‐dependent change in the extracellular DBL‐1 pathway regulators LON‐1, LON‐2, SMA‐10, CRM‐1, DRAG‐1, and ADT‐2 (Gumienny & Savage‐Dunn, 2013). Transcript abundance for *lon‐1*, *sma‐10*, and *drag‐1* was significantly changed in neural *hsf‐1* transgenic animals, but *sma‐10* was the only extracellular regulator whose transcriptional repression corresponded with *dbl‐1* levels in the cell (Figure 4b). SMA‐10, a LRIG (leucine rich and immunoglobulin domains) homolog physically binds SMA‐6 and regulates its activity (Gumienny et al., 2010). Moreover, loss of *sma‐10* aberrantly affects endocytic trafficking and reduces signal strength of the SMA‐6 receptor (Gleason et al., 2017). We hypothesized that reduced *dbl‐1* signaling by neural *hsf‐1* represses *sma‐10* expression in peripheral tissues thereby disrupting surface residency and steady‐state expression of SMA‐6 in endothelial cells, which ultimately reduces BMP signal transduction.

Since the loss of SMA‐10 disrupts the endocytic dynamics of SMA‐6 (Gleason et al., 2017), we examined other critical regulators of membrane transport and endocytosis with the potential to impact SMA‐6 surface residency. In addition to FOXO and TGF‐β signaling, KEGG pathway analysis identified a significant enrichment of transcripts differentially regulated in neural *hsf‐1* animals which were associated with endocytosis (cel04144) (Figure S2f). Of the 29 endocytosis‐annotated transcripts that were differentially regulated by neural *hsf‐1*, 27 of them were significantly repressed (Figure 4c). Therefore, reducing *dbl‐1* signaling by neural *hsf‐1* corresponded to the global repression of endocytosis‐related genes. In further support, ablation of *dbl‐1* signaling displayed a similar yet more robust repression of the same endocytic genes (Figure 4c). As critical regulators of secretory and endocytic transport (Pfeffer, 2017; Stenmark, 2009), Rab GTPases are the largest family of small G proteins within the cell (Stenmark, 2009). We observed that reducing and ablating DBL‐1 signaling in neural *hsf‐1* transgenics and *dbl‐1(nk3)* mutants resulted in approximately a 2‐fold reduction in the transcription of several Rab GTPases with roles in regulating the early endosome (RAB‐5), late endosome (RAB‐7), basolateral exocytosis (RAB‐8), and endocytic recycling (RAB‐11.1) (Figure 4c and Figure S4d). Consistent with our previous study (Watterson et al., 2022), we detected transcriptional activation of the RAB‐11.1 paralog, *rab‐11.2*, which likely occurs through loss of RAB‐11.1 expression and subsequent activation of the intracellular lipid surveillance response (Figure 4c and Figure S4d). Thus, reducing BMP/DBL‐1 signaling in the nervous system has the potential to modulate lipid surveillance and nuclear hormone receptor, NHR‐49, activity in the intestine. We confirmed NHR‐49 activation in both neural *hsf‐1* and *dbl‐1(nk3)* animals as indicated by the transcriptional induction of an established NHR‐49 target (Van Gilst, Hadjivassiliou, Jolly, et al., 2005; Van Gilst, Hadjivassiliou, & Yamamoto, 2005) in the acyl‐CoA synthase, *acs‐2* (Figure S4e). In further support, the same elevated nonpermissive temperatures, which have been reported to impact lipid metabolism genes in the intestine by neural *hsf‐1* (Chauve et al., 2021), were sufficient to activate lipid surveillance as indicated by increased fluorescence of the *rab‐11.2p::YFP* transcriptional reporter at 25°C (Figure S4f).

Due to their role in endocytic recycling and lipid surveillance, the RAB11 family of GTPases acts as critical regulators of protein residency at the apical cell surface (Welz et al., 2014). Similar to *sma‐10* mutants (Gleason et al., 2017), reducing *rab‐11.1* transcription via RNAi was sufficient to reduce cell surface residency of enteric SMA‐6::GFP with a larger portion of the fusion protein decorating FM4‐64 fluorescently‐labeled endocytic vesicles (Figure 4d). Moreover, the overall signal intensity of SMA‐6::GFP was reduced with *rab‐11.1* RNAi as evidenced by large‐particle flow cytometry (Figure 4e). In combination, reduced expression of critical regulators of membrane trafficking such as RAB‐11.1 and extracellular regulators such as SMA‐10 is sufficient to dampen BMP signaling in the peripheral tissues by reducing surface residency of the type I BMP receptor, SMA‐6.

### 2.5 SMA‐3 mediates repression of membrane trafficking

**FIGURE 5:**
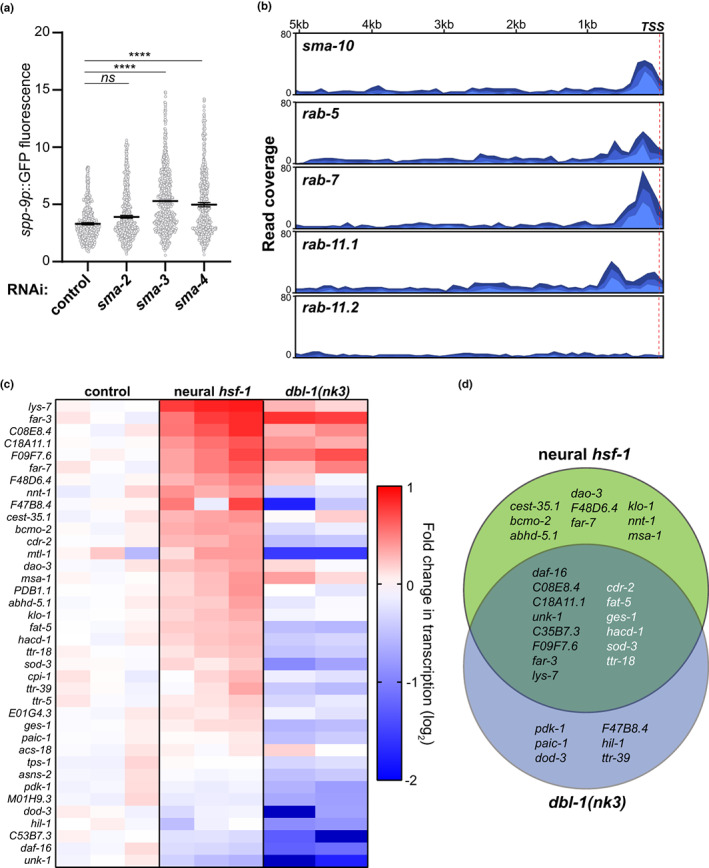
Neural *hsf‐1* represses *sma‐10* transcription through SMA‐3. (a) Relative fluorescence of Day 1 adult transgenic worms harboring the *spp‐9p*::GFP transcriptional reporter (PMD145) by large‐particle flow cytometry. Mean with 95% confidence interval, ****p ˂ 0.0001 by one‐way ANOVA with the Dunnett's multiple comparison test, *n* = 925,1340,1209,1077 from left to right over multiple independent trials. (b) Read coverage from SMA‐3 chromatin immunoprecipitations (ChIP‐seq) 5 kilobases upstream of transcriptional start sites (TSS) for the respective genes. *p* = 0.0002 (SMA‐10), *p* = 0.0597 (RAB‐5), *p* = 9.895e−10 (RAB‐7) and *p* = 0.0395 (RAB‐11.1). (c) Heat map depicts relative fold change in transcript abundance in wild‐type control (N2), neural *hsf‐1* transgenic (AGD1289), and *dbl‐1(nk3)* mutant (NU3) worms. Shown are established DAF‐16 transcriptional targets. Red = activation, blue = repression. *n* = 3, N2, AGD1289, *n* = 2, NU3. (d) Venn diagram shows the overlap of genes whose expression levels are significantly different (*p* ˂ 0.05) between the respective RNAseq datasets (multiple unpaired *t*‐tests). Genes denoted in white are transcriptionally activated in the neural *hsf‐1* and repressed in the *dbl‐1(nk3)* strains.

It remained unclear how reducing DBL‐1 signaling initiated transcriptional repression of these critical regulators of membrane trafficking and receptor dynamics. As intracellular signal transducers of DBL‐1 signaling in the periphery, SMA‐2 and SMA‐3 represent the receptor‐regulated Smads (R‐Smads) while SMA‐4 is the Co‐Smad (Savage et al., 1996) (Figure 1a). These Smad proteins engage in dynamic nucleocytoplasmic translocation with nuclear retention being stimulated by pathway activation (Inman et al., 2002). We confirmed that *sma‐3* RNAi, and to a lesser extent *sma‐4* RNAi, reduced BMP signaling as indicated by increased fluorescence of the *spp‐9p*::GFP reporter (Figure 5a). No transcriptional fluctuation was observed in neural *hsf‐1* transgenic animals for these intracellular transduction components, SMA‐2, SMA‐3, and SMA‐4 (Figure S5a). Knowing that SMA‐10 was capable of regulating SMA‐6 activity in the periphery (Gleason et al., 2017), we examined whether SMA‐3 might impact *sma‐10* transcript levels. While *sma‐6* transcription was unaffected, *sma‐3(e491)* mutant animals significantly altered the transcription of *sma‐10* (Figure S5b). Analysis of previously reported chromatin immunoprecipitations (Madaan et al., 2018) identified a SMA‐3‐binding peak within the 5′ untranslated regions immediately upstream of the *sma‐10* gene (Figure 5b). Furthermore, we observe SMA‐3‐binding peaks within the first kilobase upstream of the translation start sites for *rab‐5*, *rab‐7*, and *rab‐11.1* (Figure 5b), all of which were repressed in neural *hsf‐1* and *dbl‐1(nk3)* mutant backgrounds (Figure 4c and Figure S4d). Predicted binding sites for HSF‐1, heat shock elements, were not identified in the promoter regions of these membrane trafficking genes (Table S3). Thus, their transcriptional fluctuations are likely due to DBL‐1/BMP signal transduction in the peripheral tissues. Unlike the RAB GTPases that were repressed in neural *hsf‐1* and *dbl‐1(nk3)* mutant backgrounds, the lipid surveillance responsive transcript, *rab‐11.2*, was activated in the same worm strains without a detectable SMA‐3‐binding peak (Figure 5b). Overall, reducing receipt of the DBL‐1 signal promotes a BMP negative feedback loop within the intestine. Our data suggest that this occurs in part through SMA‐3 and its ability to repress critical factors involved in membrane trafficking and receptor dynamics, which ensure steady‐state levels and cell surface residency of the SMA‐6 receptor. However, activation of lipid surveillance under these same conditions does not appear to be directly regulated by BMP signaling and SMA‐3 but rather appears to be an indirect consequence of reduced RAB GTPase function and membrane trafficking.

### 2.6 Neural HSF‐1 does not require DAF‐16/FOXO to regulate DBL‐1 signaling

The FOXO transcription factor, DAF‐16, is required in the worm intestine for neural *hsf‐1* to extend *C. elegans* lifespan (Douglas et al., 2015). We sought to understand whether BMP signaling acts to bridge HSF‐1 activity in the nervous system with DAF‐16 in the intestine. As previously mentioned, our data indicate that reducing BMP signaling by neural *hsf‐1* resides upstream of DAF‐16 activity in the intestine (Figure 3b,c). Since DAF‐16 complexes with SMA‐3 to regulate the transcriptional output of target genes (Qi et al., 2017), we hypothesized that DAF‐16 activity converges with SMA‐3 to mediate transcriptional repression of membrane trafficking regulators. As a regulator of GTPase activity, the Ras homolog enriched in the brain, RHEB‐1, was one gene whose promoter region was reported to be co‐occupied and its expression mutually regulated by DAF‐16 and SMA‐3 (Qi et al., 2017). We confirmed SMA‐3 binding within a kilobase of the untranslated promoter region upstream of the RHEB‐1 translational start site (Figure S5c) and this corresponded to reduced *rheb‐1* transcription in both neural *hsf‐1* and *dbl‐1(nk3)* mutant strains (Figure S5d). Thus, a previously characterized promoter mutually occupied by both DAF‐16 and SMA‐3 showed repression of its respective transcript upon reduction and ablation of *dbl‐1* signaling.

While we expect similar transcriptional signatures between reduced *dbl‐1* signaling by neural *hsf‐1* and signal ablation via *dbl‐1(nk3)* mutants, we anticipate select differences, which likely account for their longevity effects. To investigate this, we compared a core set of 37 *daf‐16* transcriptional targets, which were compiled across several studies (Kumar et al., 2015; McElwee et al., 2003; Murphy et al., 2003; Riedel et al., 2013), and observed significant changes in 62% of these core transcripts in neural *hsf‐1* animals while *dbl‐1(nk3)* null mutations modulated 54% of these core genes (Figure 5c,d). Select *daf‐16* targets including *fat‐5*, *cdr‐2*, and *hacd‐1* with subtle transcriptional changes were confirmed by qPCR (Figure S5e). While neural *hsf‐1* transgenic animals tended to exhibit stronger activation of these core *daf‐16* targets, the *dbl‐1(nk3)* mutants had a more repressive effect. Of the six DAF‐16 transcriptional targets, which were significantly activated in neural *hsf‐1* transgenics and repressed in *dbl‐1(nk3)*, the carboxylesterase, GES‐1, the stearoyl‐CoA desaturase, FAT‐5, and the superoxide dismutase, SOD‐3, have been implicated in previous aging studies (Honda & Honda, 1999; Libina et al., 2003; Murphy et al., 2003). Furthermore, the hydroxyacyl‐CoA dehydrogenase, HACD‐1, in addition to GES‐1 and FAT‐5 play important roles in lipid metabolism while SOD‐3 and the cadmium‐responsive, GST‐domain containing, CDR‐2, protect against cellular redox imbalances, which are closely linked with mitochondrial energetics and metabolic output. It is possible that this subset of genes with strong metabolic links are acting through lipid surveillance mechanisms to impact age determination via neural *hsf‐1*.

Overall, our studies support a model in which neural *hsf‐1* regulates DBL‐1 ligand production in the nervous system while reducing signal transmission has the potential to extend animal lifespan by driving a negative feedback loop in the BMP/DBL‐1 signaling pathway through SMA‐3 and activating intracellular lipid surveillance. Dampening BMP/DBL‐1 signaling is further enhanced by the reduced expression of small G proteins involved in membrane transport and the extracellular regulator, SMA‐10, which maintains the SMA‐6 receptor at the basolateral surfaces of intestinal epithelia.

## 3 DISCUSSION

Herein, we provide molecular details underlying a transcellular signaling paradigm involving the heat shock transcription factor, HSF‐1, in the nervous system and its ability to modulate animal physiology and age progression across tissues through the TGF‐β/BMP signaling pathway. Previous studies have identified that HSF1 activity in cancer‐associated fibroblasts promotes malignancy through two stromal‐derived signaling molecules; TGF‐β and the stromal‐derived factor, SDF1 (Scherz‐Shouval et al., 2014). More recently in *C. elegans*, it was shown that *hsf‐1* overexpression in the nervous system can reduce BMP/DBL‐1 signaling in peripheral tissues (Chauve et al., 2021). Our studies highlight BMP/DBL‐1 signaling as a means by which neural *hsf‐1* extends animal lifespan and places this signal transduction mechanism upstream of the FOXO transcription factor, DAF‐16. Specifically, we postulate that neural *hsf‐1* represses *dbl‐1* expression through binding to consensus heat shock elements within its 5′ promoter region. Decreasing neural expression of *dbl‐1* reduces BMP signal transduction in intestinal epithelia, in part, through loss of SMA‐6 receptor availability at the cell surface, which occurs through SMA‐3‐mediated repression of *sma‐10* and several Rab GTPases. Previous studies have demonstrated that downstream nucleocytoplasmic SMAD proteins, SMA‐3 and SMA‐2, directly interact with DAF‐16 (Qi et al., 2017) and provide a likely point of convergence between BMP signaling and DAF‐16‐mediated age regulation. While previous studies report a modest lifespan extension in *dbl‐1(nk3)* mutants (Luo et al., 2009), removal of *dbl‐1* alone was not sufficient to extend lifespan in our experimental paradigm but was required for neural *hsf‐1* to extend lifespan. We hypothesize that in addition to reducing *dbl‐1* signaling, transcriptional fluctuations observed in neural *hsf‐1* and not in *dbl‐1(nk3)* null mutants alter critical molecular pathways, which are needed in addition to reduced BMP/DBL‐1 signaling to prolong aging. Knowing that DAF‐16 is also required for lifespan extension by neural *hsf‐1* (Douglas et al., 2015) yet its overexpression has been reported to yield modest lifespan extensions (Henderson & Johnson, 2001), perhaps both reduced DBL‐1 signaling and DAF‐16 activation work synergistically to provide robust lifespan effects. However, additional studies are required to further understand how BMP/DBL‐1 signaling impacts the molecular dynamics of FOXO within the nucleus.

BMP signaling plays an important role in growth and development and thus has implications in several human disease states (Wang et al., 2014). Yet reducing DBL‐1/BMP activity in *C. elegans* and subsequently restricting growth through development possesses age‐defying properties (Luo et al., 2009). This is reminiscent of another well‐established age‐extension paradigm in reduced insulin signaling (Kenyon et al., 1993), which also requires DAF‐16 expression in the intestine (Libina et al., 2003; Uno et al., 2021). In both cases, whether it be insulin or BMP signaling, restricting but not ablating its signaling capacity can extend the lifespan in a FOXO‐dependent manner. Consistent with our observation regarding age regulation by neural *hsf‐1*, BMP signaling acts upstream of insulin signaling and DAF‐16 in the context of lipid metabolism (Clark et al., 2018, 2021). Recent studies have shown that neural *hsf‐1* regulates metabolic genes, which control fatty acid saturation and thus have the capacity to impact membrane fluidity and overall lipid availability in the intestine (Chauve et al., 2021), the major site of lipid storage in the worm (Ashrafi et al., 2003). Perhaps similar to the disposable soma theory of aging (Kirkwood & Holliday, 1979), restricting organismal growth and development earlier in life as observed in neural *hsf‐1* transgenics might enable these animals to reinvest their molecular resources and metabolic reserves later in life. Yet the same metabolic genes regulated by neural *hsf‐1* overexpression (Chauve et al., 2021) were also reported to be similarly modulated upon *hsf‐1* RNAi treatments (Brunquell et al., 2016). Thus, further studies are required to understand this inexplicable link between HSF‐1, BMP signaling, and lipid metabolism. While our studies focused on the gut–neuron axis, BMP signaling to other tissues yields alternate physiological consequences as demonstrated by BMP/DBL‐1 signaling to the hypodermis, which is an important determinant in animal body length (Wang et al., 2002). Our studies show that rescuing SMA‐6 expression exclusively in the intestine of the short‐lived *sma‐6(wk7)* enables neural *hsf‐1* to extend lifespan. This is consistent with the requirement of DAF‐16 in the intestine for neural *hsf‐1* to extend animal lifespan (Douglas et al., 2015). Yet in both cases, a lifespan extension typical of neural *hsf‐1* was not fully achieved by the enteric rescue of either SMA‐6 or DAF‐16, suggesting that signaling between other tissues like the hypodermis or the germline, likely plays a complementary role in age regulation by the heat shock factor.

Our data suggest that HSF‐1 in the nervous system communicates to DAF‐16 in the intestine through BMP/DBL‐1 signaling. Previous studies have demonstrated that the homeobox domain protein, CEH‐28, activates DBL‐1 expression specifically in M4 neurons (Ramakrishnan et al., 2014). Our data provide additional regulatory information regarding the controlled expression of this DBL‐1 ligand as HSF‐1 appears to bind consensus heat shock elements in the 5′ promoter region of DBL‐1. It remains unclear whether HSF‐1 acts in these M4 neurons in conjunction with CEH‐28 to modulate DBL‐1 expression or whether it is acting in other parts of the nervous system to repress *dbl‐1* expression and promote longevity. Upon receipt of the DBL‐1 ligand in peripheral tissues, signal transduction ultimately leads to the nuclear accumulation of SMA‐2/SMA‐3 (Gumienny & Savage‐Dunn, 2013), which can co‐occupy some of the same DNA regulator elements as DAF‐16 (Qi et al., 2017). We observe the SMA‐10 and several RAB GTPases are significantly repressed by neural *hsf‐1* and in *dbl‐1* mutants. Consistent with the removal of *dbl‐1* being sufficient to repress transcription of these genes, we observed SMA‐3 binding to the promoter regions of these membrane trafficking genes. Thus, under times of growth and development, DBL‐1 would likely promote the expression of these genes to help establish polarity and maximize nutrient uptake through ensured cell surface residency of nutrient transport machinery. While neural *hsf‐1* reduces *dbl‐1* expression and extends animal lifespan, *dbl‐1* ablation does not extend animal lifespan in our laboratory, yet it still acts to repress transcription of *sma‐10* and several Rab GTPases, and reduce steady‐state levels of intestinal SMA‐6::GFP. Thus, further characterization of nuclear interactions between SMA‐3 and DAF‐16 might help identify additional factors that contribute to lifespan extension through reduced DBL‐1 signaling.

## 4 METHODS

### 4.1 *Caenorhabditis elegans* strains and maintenance

Worm strains were maintained at 15°C on an OP50 *E. coli* lawn grown on nematode growth medium (NGM) plates and all experiments were performed at 20°C unless otherwise noted in the text. Age synchronization was performed by treatment with hypochlorite of gravid animals to obtain eggs. The following strains were obtained from the Caenorhabditis Genetics Center (CGC): N2 (wild‐type (WT)), CF512 (*rrf‐3(b26) II; fem‐1(hc17) IV*), NU3 (*dbl‐1(nk3)V*), LT186 (*sma‐6(wk7)II*), CB491 (*sma‐3(e491)III*), LW2436 (*RAD‐SMAD*: jjIs2277 [pCXT51(5*RLR::pes‐10p(deleted)::GFP) + LiuFD61(mec‐7p::RFP)]; I or IV).

The following strains were obtained from external laboratories: AGD1289 (neural hsf‐1 FL: *uthIs368[rab‐3p::HSF‐1 FL*, *myo‐2p::tomato]*) worm strain was obtained from the laboratory of Andrew Dillin. LT620 (*wkEx52 [spp‐9p::GFP]*) was created by the Richard Padget group. RT2495 (*unc‐119(ed3); pwIs921[vha‐6p::SMA‐6::GFP]*) was created by the Barth Grant group.

The following strains were generated for this study: PMD22 (*dbl‐1(nk3)V; uthIS368[rab‐3p::HSF‐1 FL*, *myo‐2p::tomato] strain*) by crossing strain NU3 with AGD1289, PMD23 (*sma‐6(wk7)II; uthIs368[rab‐3p::HSF‐1 FL*, *myo‐2p::tomato] strain*) by crossing strain LT186 with AGD1289, PMD145 (*wkIs52(spp‐9p::GFP); integrated on X chromosome*) by integrating strain LT620, PMD93 (*spp‐9p::gfp (integrated); uthIS366[rab‐3p::HSF‐1 FL*, *myo‐2p::tomato] strain*) by crossing strain PMD145 with AGD1289, PMD79 (*uthIs368[rab‐3p::HSF‐1 FL*, myo‐2p::tomato] strain; jjIs2277[pCXT51(5*RLR::pes‐10p(deleted)::GFP) + LiuFD61(mec‐7p::RFP)]; integrated on LG I or V; RAD‐SMAD), PMD35 (*vha‐6p::SMA‐6::GFP; uthIS368[rab‐3p::HSF‐1 FL*, *myo‐2p::tomato] strain*) by crossing strain RT2495 with AGD1289, PMD87 (*vha‐6p::SMA‐6::GFP; dbl‐1(nk3)*) by crossing strain RT2495 with NU3, PMD74 (*vha‐6p::SMA‐6::GFP; sma‐6(wk7)*) by crossing strain RT2495 with LT186, PMD69 (*uthIS368[rab‐3p::HSF‐1 FL*, *myo‐2p::tomato] strain; sma‐6(wk7); vha‐6p::SMA‐6::GFP (intestine)*), PMD144 (*wkEx52 [spp‐9p::GFP (extrachromosomal)]*), PMD74 (*wkEx52 [spp‐9p::GFP (extrachromosomal)]; uthIS368[rab‐3p::HSF‐1 FL*, *myo‐2p::tomato] strain*) by crossing strain PMD144 with AGD1289. PMD64 (*wkEx52 [spp‐9p::gfp]; uthIS368[rab‐3p::HSF‐1 FL*, *myo‐2p::tomato] strain*).

### 4.2 RNAi administration

RNA interference experiments were performed using RNAi strains acquired from *C. elegans* RNAi feeding libraries created by Ahringer and Vidal. Each library consists of HT115 *E. coli* containing the L4440 empty vector (EV) plasmid that houses various RNAi constructs for the knockdown of specific genes. Within this study, HT115 *E. coli* containing the L4440 empty vector (EV) was used as control versus other various RNAi constructs. RNAi was initially cultured in liquid Terrific Broth (TB) for 14–16 h at 37°C then induced with 1 mM IPTG for 4 h at 37°C. Bacterial cultures were concentrated 1/10 via centrifugation at 4400 *g* for 10 min, seeded onto RNAi plates (60 mm or 100 mm, NGM containing 100 μg/ml carbenicillin and 1 mM IPTG added 24 h prior to seeding), and allowed to dry for 1–2 days at room temperature, protected from light.

### 4.3 *Caenorhabditis elegans* lifespan analysis

Worms were age‐synchronized using hypochlorite treatment of gravid animals for egg retrieval. Eggs were transferred onto RNAi plates seeded with EV control or different RNAi constructs. 100 μg/ml 5‐fluorouracil‐2′‐deoxyribose (FUdR) was supplemented to media plates on Day 1 of adulthood to avoid progeny that would disrupt the synchronization of the worm population. Each lifespan was set up to consist of 10 plates per condition, with at least 10 worms per plate, and at least 100 worms per condition. Plates were scored every day or every other day for “censored” or “dead” worms. Worms that were censored were those that were missing from the plate, bagged with progeny, exploded/ruptured, or desiccated. Whole plates were only censored in the event of fungal/bacterial contamination. Worms that showed no response to touch with a platinum wire at the head or tail were scored as dead. To alleviate transgenic silencing observed in neural *hsf‐1* transgenic animals (AGD1289), worms were thawed from frozen stocks and allowed to recover for 3–4 generations prior to lifespan analysis.

### 4.4 Quantitative PCR

Quantitative reverse‐transcriptase PCR (qPCR) was performed as previously described Egge et al. 2019. Worms were age‐synchronized by hypochlorite treatment and grown on EV control or experimental RNAi at 20°C until the L4 stage of development. Total RNA was extracted by three rounds of freeze/thaw using TRIzol (ThermoFisher Scientific), followed by a chloroform and isopropanol precipitation extraction process. RNA pellets were rinsed twice with 75% ethanol, air‐dried, and re‐suspended in 20–50 μl molecular biology grade water. A DS‐11 FX+ spectrometer (DeNovix) was used to measure RNA concentration including 260/280 and 260/230 ratios. Reverse transcription was conducted using the QuantiTect Reverse Transcription kit (Qiagen). 1 μg of RNA was used to synthesize cDNA according to the manufacturer's guidelines. For qPCR, 20 μl reactions were performed using the iTaq™ Universal SYBR® Green Supermix kit (Bio‐Rad) with 5 ng cDNA per well in a CFX384 Real Time System (Bio‐Rad). Each sample was loaded in technical triplicate, and three biological replicates were analyzed for each condition. The relative transcript levels for each target gene and two housekeeping genes, *tba‐1* and Y45F10D.4, were calculated with the ∆Ct method. The geometric mean of the two housekeeping transcripts was used to normalize target gene expression. The following primers were used for qPCR analysis:

*hsf‐1* FWD: 5′‐TCAGACAGTTGAATATGTACGG‐3′
*hsf‐1* REV: 5′‐CCTGATCTGATTCTGTTCGAG‐3′
*dbl‐1* FWD: 5′‐ACCTTGCTGTGTGCCTACTG‐3′
*dbl‐1* REV: 5′‐CCGCATGTCGGCATACTCTC‐3′
*daf‐7* FOR: 5′‐CTTCTTTCCCTCCCACATGGT‐3′
*daf‐7* REV: 5′‐AGGTACTCTGTTCGGTGCTG‐3′
*spp‐9* FOR: 5′‐GTTGAAGATAAATTCCTTGCCG‐3′
*spp‐9* REV: 5′‐GGCGTAGTTTAGACAAGTCTG‐3′
*sma‐6* FWD: 5′‐AGGCTGCGTTGATCCAAAGG‐3′
*sma‐6* REV: 5′‐TACAGCCACGTAACTTCCACG‐3′
*sma‐10* FWD: 5′‐AGTTTGGCAAGAAACGACGTG‐3′
*sma‐10* REV: 5′‐ATCCAACCTTCCGTAACCGC‐3′

All amplifications were performed at 95°C for 1 min, followed by 40 cycles at 95°C for 15 s, 60°C for 1 min, and a melt curve analysis consisting of 60 cycles at 65–95°C, 0.5°C/cycle (increment), 0.5°C/s (ramp).

### 4.5 Protein extraction and western blotting

All western blots were performed on late L4 larvae/day 1 adult worms. Animals have washed off the plates with liquid M9 buffer, centrifuged at 1000 *g* for 30 s, and washed twice with M9 before being transferred to 1.5 ml Eppendorf tubes and rapidly flash frozen in liquid nitrogen. Frozen worm pellets were thawed on ice and worm extracts were generated by glass/zirconia bead disruption in nondenaturing lysis buffer (50 mM Hepes pH 7.4, 150 mM NaCl, 1 mM EDTA, 1% Triton, EDTA‐free mini‐protease inhibitor cocktail [Roche]). Crude lysates were subject to centrifugation at 8000 *g* at 4°C for 5 min prior to protein determination with a BCA protein quantification kit (Thermo Scientific). Lysates were supplemented with 2× Laemmli sample buffer, boiled at 90°C for 10 min, resolved by SDS‐PAGE, transferred to 0.22 μm nitrocellulose membranes, and subject to western blot analysis (Egge et al., 2019). Electrophoresis was performed at a constant 100 V and protein size was compared with the prestained protein ladder (EZ‐RUN, ThermoFisher).

All antibodies were prepared in 5% BSA/PBST. The membrane was incubated with primary antibody overnight at 4°C followed by the secondary antibody for 1 hour at room temperature. Rabbit anti‐α‐Tubulin (Cat. No. ab4074, Abcam) primary antibody was used at 1:15,000, and secondary anti‐IgG goat polyclonal antibody horseradish peroxidase (Cat. No. 20403‐1ML, Biotium) was used at 1:5000. Both the rabbit anti‐GFP antibody (ThermoFisher, A6455) and the anti‐IgG goat polyclonal antibody horseradish peroxidase (Cat. No. 20403‐1ML, Biotium) were used at 1:5000. Bands were visualized after incubation with Clarity Western ECL Substrate (Bio‐Rad, 1705061) and detection on film was performed using a Konica Minolta SRX‐101A film processor. Samples were run in triplicate and ratioed band intensities were averaged for each biological replicate.

### 4.6 Microscopy

For representative fluorescence micrographs of transcriptional reporter strains, five to six worms per condition were aligned on NGM/carb plates in a drop of M9 supplemented with 100 mM levamisole. Imaging was performed on a Zeiss Axio Zoom.V16 set to 100× magnification. Images were acquired using transmitted light and standard filter settings for excitation and emission of fluorescence probes and recorded on a CCD camera (Zeiss AxioCam 503 mono). Zeiss ZEN software was used to control acquisition. Exposure settings and additional processing parameters remained consistent among samples in each experiment.

To examine SMA‐6::GFP co‐localization with FM4‐64, approximately 100 days, 1 adult worms were rinsed off plates with M9 buffer, centrifuged (1000 *g*) for 30 s, and washed twice with M9 buffer. Worms were then incubated in 100 μl 0.4 mM FM4‐64 (Fisher Scientific, T3166) for 2 h at 20°C in an Eppendorf Thermomixer C shaker set to 300 rpm. Following incubation, worms were washed three times with M9 and allowed to recover on NGM plates seeded with OP50 for approximately 30 min in the dark. Confocal micrographs were acquired using a Leica SP8 confocal microscope (Leica, Buffalo Grove, IL) and Leica Application Suite X (LAS X) software. Live worms were mounted in M9 supplemented with 100 mM levamisole and samples were imaged at 40× or 63× with oil immersion. All images were acquired in xyz acquisition mode at a speed of 600, and each line was imaged three times and averaged to reduce noise. Lasers 488 and/or 552 were used to excite fluorophores using hybrid detectors (HyD). Laser power, range, and gain were adjusted according to strain/experiment but remained consistent between independent repeats.

### 4.7 Large‐particle flow cytometry

A COPAS FP‐250 flow cytometer (Union Biometrica) was used to analyze age‐synchronized worms using the attached sample cup or acquired from a 96‐well plate by an LP Sampler (Union Biometrica). Sample solution was comprised of M9 while the flow sheath solution contained a proprietary recipe, COPAS GP sheath reagent (PN: 300‐5070‐100, Union Biometrica). Flow data were collected and processed using the FlowPilot software (ver. 2.6.1, Union Biometrica). Further data processing was performed in Excel (Microsoft) and statistical analysis in Prism 9 (Graphpad). Extinction was detected using the 488 nm laser line with a 1.3 ND filter and the gain of 1.0. Propidium iodide, mCherry, and DsRed were excited using a 561 nm laser, while YFP and GFP were excited using a 488 nm laser. Gains for fluorescence detection were set to 2.0, and PMT voltage adjusted within the linear range of the instrument was consistent for each experiment (500 for YFP, mCherry, DsRed, and 450 for GFP). WormProfiler (an in‐house program written for the MATLAB runtime) was used to standardize worm lengths and calculate fluorescence intensity profiles for individual worms within samples. Unless otherwise noted, the integral of the fluorescence intensity for each worm within samples was averaged, and mean values were normalized according to time‐of‐flight (TOF) to account for variation in worm size across RNAi conditions. Mean integral or peak fluorescent values were standardized relative to empty vector control.

### 4.8 Transcriptomics and bioinformatics

Three biological repeats of age‐synchronized wild‐type, neural *hsf‐1* (AGD1289), *dbl‐1(nk3)* (NU3), and neural *hsf‐1*;*dbl‐1(nk3)* (PMD22) worms cultured on HT115 *E. coli* until late L4 larval stages were collected and rapidly flash frozen in liquid nitrogen. RNA was purified by chloroform/phenol extraction followed by isopropanol precipitation and two washes with 75% ethanol before resuspension in 50 μl molecular biology grade water. Quality control, mRNA purification, and paired‐end 150 bp Illumina sequencing were performed by Novogene as previously described (Egge et al., 2021). Statistical analysis was performed using CLC software (version 9.5, CLC Bio). Gene expression data were compared by the Baggerly's test and genes were considered significantly regulated when absolute fold change ≥1.5 and *p*‐value ˂0.05. Deposited ChIP‐seq datasets from the previous publication were analyzed using the CLC genomic workbench software v9.5. Raw data obtained from either Geo Datasets (GSE81523 for HSF‐1::GFP ChIP‐seq) or EnCode datasets (ENCSR992FVB for SMA‐3::GFP ChIP‐seq) were trimmed and mapped to the reference *C. elegans* genome (NCBI accession number NC_002944). With respect to HSF‐1 binding within the DBL‐1 promoter in a subset of neurons, genomic reads isolated in complex with HSF‐1::GFP were divided by the total input reads for the same respective genomic region. The CLC shape‐based peak caller was used for all SMA‐3::GFP ChIP. ChIP‐enriched DNA was aligned onto input DNA; when the sequence coverage of a genomic region in the enriched DNA exceeded the input DNA, a ChIP peak score was called. ChIP peaks with their respective P values determined by CLC were generated for candidates whose transcript levels were differentially regulated as determined by RNAseq. To identify heat shock element consensus motif presence in the 5′ untranslated promoter region of the DBL‐1 gene, we examined 2.5 kilobases upstream of the DBL‐1 translation start site using the MEME suite (v4.11.1) (Bailey et al., 2015). Additional analysis for HSEs within the promoters of other genes was determined using TFBIND (https://tfbind.hgc.jp/). For each gene, 1000 bp upstream of the TSS (translational start site) was searched for sites with >90% sequence similarity of the HSF‐1 consensus binding site, obtained from the transcription factor matrix ID (TRANSFAC R.3.4), on both the (+) and (−) strand.

### 4.9 Statistical analysis

Statistical analyses, including t‐test, mixed‐effects, Chi‐squared, and ANOVA with post hoc multiple comparisons analysis, were conducted using Prism (version 9.0, GraphPad) and statistical analysis for large transcriptomic datasets was performed using CLC genomics workbench (version 9.5). For large worm populations, outliers were removed using the ROUT method (*Q* = 1%) prior to analysis.

## AUTHOR CONTRIBUTIONS

S.L.B.A., J.M., and P.M.D. involved in conceptualization; S.L.B.A., J.M., L.T., A.W., K.R.Z., B.M., T.L.G., and P.M.D. involved in methodology; S.L.B.A., J.M., L.T., A.W., K.R.Z., B.M., T.L.G., and P.M.D. involved in investigation; S.L.B.A., T.L.G., and P.M.D. involved in writing—review and edit; T.L.G. and P.M.D. involved in funding acquisition, resources, and supervision.

## CONFLICT OF INTEREST

The authors declare no competing interests.

## Supporting information


Figure S1

Figure S2

Figure S3

Figure S4

Figure S5

Table S1

Table S2

Table S3

## Data Availability

All data generated and analyzed during this study are included in this article and its extended data are also available from the authors upon reasonable request. Transcriptomic data files that support the findings of this study in *C. elegans* will be deposited in the NCBI Gene Expression Omnibus (GEO) upon publication.

## References

[acel13693-bib-0001] Akerfelt, M. , Morimoto, R. I. , & Sistonen, L. (2010). Heat shock factors: Integrators of cell stress, development and lifespan. Nature Reviews. Molecular Cell Biology, 11, 545–555. 10.1038/nrm2938 20628411 PMC3402356

[acel13693-bib-0002] Ambrosini, Y. M. , Borcherding, D. , Kanthasamy, A. , Kim, H. J. , Willette, A. A. , Jergens, A. , Allenspach, K. , & Mochel, J. P. (2019). The gut‐brain axis in neurodegenerative diseases and relevance of the canine model: A review. Frontiers in Aging Neuroscience, 11, 130. 10.3389/fnagi.2019.00130 31275138 PMC6591269

[acel13693-bib-0003] Ashrafi, K. , Chang, F. Y. , Watts, J. L. , Fraser, A. G. , Kamath, R. S. , Ahringer, J. , & Ruvkun, G. (2003). Genome‐wide RNAi analysis of *Caenorhabditis elegans* fat regulatory genes. Nature, 421, 268–272. 10.1038/nature01279 12529643

[acel13693-bib-0004] Bailey, T. L. , Johnson, J. , Grant, C. E. , & Noble, W. S. (2015). The MEME suite. Nucleic Acids Research, 43, W39–W49. 10.1093/nar/gkv416 25953851 PMC4489269

[acel13693-bib-0005] Boehme, M. , van de Wouw, M. , Bastiaanssen, T. F. S. , Olavarría‐Ramírez, L. , Lyons, K. , Fouhy, F. , Golubeva, A. V. , Moloney, G. M. , Minuto, C. , Sandhu, K. V. , Scott, K. A. , Clarke, G. , Stanton, C. , Dinan, T. G. , Schellekens, H. , & Cryan, J. F. (2020). Mid‐life microbiota crises: Middle age is associated with pervasive neuroimmune alterations that are reversed by targeting the gut microbiome. Molecular Psychiatry, 25, 2567–2583. 10.1038/s41380-019-0425-1 31092898

[acel13693-bib-0006] Brunquell, J. , Morris, S. , Lu, Y. , Cheng, F. , & Westerheide, S. D. (2016). The genome‐wide role of HSF‐1 in the regulation of gene expression in *Caenorhabditis elegans* . BMC Genomics, 17, 559. 10.1186/s12864-016-2837-5 27496166 PMC4975890

[acel13693-bib-0007] Carabotti, M. , Scirocco, A. , Maselli, M. A. , & Severi, C. (2015). The gut‐brain axis: Interactions between enteric microbiota, central and enteric nervous systems. Annals of Gastroenterology, 28, 203–209. 25830558 PMC4367209

[acel13693-bib-0008] Chauve, L. , Hodge, F. , Murdoch, S. , Masoudzadeh, F. , Mann, H. J. , Lopez‐Clavijo, A. F. , Okkenhaug, H. , West, G. , Sousa, B. C. , Segonds‐Pichon, A. , Li, C. , Wingett, S. W. , Kienberger, H. , Kleigrewe, K. , de Bono, M. , Wakelam, M. J. O. , & Casanueva, O. (2021). Neuronal HSF‐1 coordinates the propagation of fat desaturation across tissues to enable adaptation to high temperatures in *C. elegans* . PLoS Biology, 19, e3001431. 10.1371/journal.pbio.3001431 34723964 PMC8585009

[acel13693-bib-0009] Clark, J. F. , Ciccarelli, E. J. , Kayastha, P. , Ranepura, G. , Yamamoto, K. K. , Hasan, M. S. , Madaan, U. , Meléndez, A. , & Savage‐Dunn, C. (2021). BMP pathway regulation of insulin signaling components promotes lipid storage in Caenorhabditis elegans. PLoS Genetics, 17, e1009836. 10.1371/journal.pgen.1009836 34634043 PMC8530300

[acel13693-bib-0010] Clark, J. F. , Meade, M. , Ranepura, G. , Hall, D. H. , & Savage‐Dunn, C. (2018). *Caenorhabditis elegans* DBL‐1/BMP regulates lipid accumulation via interaction with insulin signaling. G3: Genes, Genomes, Genetics, 8, 343–351. 10.1534/g3.117.300416 29162682 PMC5765361

[acel13693-bib-0011] Douglas, P. M. , Baird, N. A. , Simic, M. S. , Uhlein, S. , McCormick, M. A. , Wolff, S. C. , Kennedy, B. K. , & Dillin, A. (2015). Heterotypic signals from neural HSF‐1 separate thermotolerance from longevity. Cell Reports, 12, 1196–1204. 10.1016/j.celrep.2015.07.026 26257177 PMC4889220

[acel13693-bib-0012] Durieux, J. , Wolff, S. , & Dillin, A. (2011). The cell‐non‐autonomous nature of electron transport chain‐mediated longevity. Cell, 144, 79–91. 10.1016/j.cell.2010.12.016 21215371 PMC3062502

[acel13693-bib-0013] Egge, N. , Arneaud, S. L. B. , Fonseca, R. S. , Zuurbier, K. R. , McClendon, J. , & Douglas, P. M. (2021). Trauma‐induced regulation of VHP‐1 modulates the cellular response to mechanical stress. Nature Communications, 12, 1484. 10.1038/s41467-021-21611-8 PMC7935884 33674585

[acel13693-bib-0014] Egge, N. , Arneaud, S. L. B. , Wales, P. , Mihelakis, M. , McClendon, J. , Fonseca, R. S. , Savelle, C. , Gonzalez, I. , Ghorashi, A. , Yadavalli, S. , Lehman, W. J. , Mirzaei, H. , & Douglas, P. M. (2019). Age‐onset phosphorylation of a minor actin variant promotes intestinal barrier dysfunction. Developmental Cell, 51, 587–601e7. 10.1016/j.devcel.2019.11.001 31794717 PMC6897307

[acel13693-bib-0015] Gleason, R. J. , Vora, M. , Li, Y. , Kane, N. S. , Liao, K. , & Padgett, R. W. (2017). *C. elegans* SMA‐10 regulates BMP receptor trafficking. PLoS One, 12, e0180681. 10.1371/journal.pone.0180681 28704415 PMC5509155

[acel13693-bib-0062] Gumienny, T. L. , MacNeil, L. T. , Wang, H. , de Bono, M. , Wrana, J. L. , & Padgett, R. W. (2007). Glypican LON‐2 is a conserved negative regulator of BMP‐like signaling in Caenorhabditis elegans. Current Biology., 17(2), 159–164. 17240342 10.1016/j.cub.2006.11.065

[acel13693-bib-0016] Gumienny, T. L. , MacNeil, L. , Zimmerman, C. M. , Wang, H. , Chin, L. , Wrana, J. L. , & Padgett, R. W. (2010). Caenorhabditis elegans SMA‐10/LRIG is a conserved transmembrane protein that enhances bone morphogenetic protein signaling. PLoS Genetics, 6, e1000963. 10.1371/journal.pgen.1000963 20502686 PMC2873917

[acel13693-bib-0017] Gumienny, T. L. , & Savage‐Dunn, C. (2013). TGF‐beta signaling in *C. elegans* . WormBook, 1‐34, 1–34. 10.1895/wormbook.1.22.2 PMC5081272 23908056

[acel13693-bib-0018] Henderson, S. T. , & Johnson, T. E. (2001). daf‐16 integrates developmental and environmental inputs to mediate aging in the nematode *Caenorhabditis elegans* . Current Biology, 11, 1975–1980. 10.1016/s0960-9822(01)00594-2 11747825

[acel13693-bib-0019] Honda, Y. , & Honda, S. (1999). The daf‐2 gene network for longevity regulates oxidative stress resistance and Mn‐superoxide dismutase gene expression in *Caenorhabditis elegans* . The FASEB Journal, 13, 1385–1393. 10428762

[acel13693-bib-0020] Hsu, A. L. , Murphy, C. T. , & Kenyon, C. (2003). Regulation of aging and age‐related disease by DAF‐16 and heat‐shock factor. Science, 300, 1142–1145. 10.1126/science.1083701 12750521

[acel13693-bib-0021] Inman, G. J. , Nicolas, F. J. , & Hill, C. S. (2002). Nucleocytoplasmic shuttling of Smads 2, 3, and 4 permits sensing of TGF‐beta receptor activity. Molecular Cell, 10, 283–294. 10.1016/s1097-2765(02)00585-3 12191474

[acel13693-bib-0022] Jena, A. , Montoya, C. A. , Mullaney, J. A. , Dilger, R. N. , Young, W. , McNabb, W. C. , & Roy, N. C. (2020). Gut‐brain axis in the early postnatal years of life: A developmental perspective. Frontiers in Integrative Neuroscience, 14, 44. 10.3389/fnint.2020.00044 32848651 PMC7419604

[acel13693-bib-0023] Kaplan, R. E. , Chen, Y. , Moore, B. T. , Jordan, J. M. , Maxwell, C. S. , Schindler, A. J. , & Baugh, L. R. (2015). dbl‐1/TGF‐beta and daf‐12/NHR signaling mediate cell‐nonautonomous effects of daf‐16/FOXO on starvation‐induced developmental arrest. PLoS Genetics, 11, e1005731. 10.1371/journal.pgen.1005731 26656736 PMC4676721

[acel13693-bib-0024] Kenyon, C. , Chang, J. , Gensch, E. , Rudner, A. , & Tabtiang, R. (1993). A *C. elegans* mutant that lives twice as long as wild type. Nature, 366, 461–464. 10.1038/366461a0 8247153

[acel13693-bib-0025] Kenyon, C. J. (2010). The genetics of ageing. Nature, 464, 504–512. 10.1038/nature08980 20336132

[acel13693-bib-0026] Kirkwood, T. B. , & Holliday, R. (1979). The evolution of ageing and longevity. Proceedings of the Royal Society of London. Series B: Biological Sciences, 205, 531–546. 42059 10.1098/rspb.1979.0083

[acel13693-bib-0027] Kumar, N. , Jain, V. , Singh, A. , Jagtap, U. , Verma, S. , & Mukhopadhyay, A. (2015). Genome‐wide endogenous DAF‐16/FOXO recruitment dynamics during lowered insulin signalling in *C. elegans* . Oncotarget, 6, 41418–41433. 10.18632/oncotarget.6282 26539642 PMC4747164

[acel13693-bib-0028] Labbadia, J. , & Morimoto, R. I. (2015). Repression of the heat shock response is a programmed event at the onset of reproduction. Molecular Cell, 59, 639–650. 10.1016/j.molcel.2015.06.027 26212459 PMC4546525

[acel13693-bib-0029] Libina, N. , Berman, J. R. , & Kenyon, C. (2003). Tissue‐specific activities of *C. elegans* DAF‐16 in the regulation of lifespan. Cell, 115, 489–502. 14622602 10.1016/s0092-8674(03)00889-4

[acel13693-bib-0030] Liu, H. , Zhang, R. , & Wang, D. (2020). Response of DBL‐1/TGF‐beta signaling‐mediated neuron‐intestine communication to nanopolystyrene in nematode *Caenorhabditis elegans* . Science of the Total Environment, 745, 141047. 10.1016/j.scitotenv.2020.141047 32758726

[acel13693-bib-0031] Luo, S. , Kleemann, G. A. , Ashraf, J. M. , Shaw, W. M. , & Murphy, C. T. (2010). TGF‐beta and insulin signaling regulate reproductive aging via oocyte and germline quality maintenance. Cell, 143, 299–312. 10.1016/j.cell.2010.09.013 20946987 PMC2955983

[acel13693-bib-0032] Luo, S. , Shaw, W. M. , Ashraf, J. , & Murphy, C. T. (2009). TGF‐beta Sma/Mab signaling mutations uncouple reproductive aging from somatic aging. PLoS Genetics, 5, e1000789. 10.1371/journal.pgen.1000789 20041217 PMC2791159

[acel13693-bib-0033] Madaan, U. , Yzeiraj, E. , Meade, M. , Clark, J. F. , Rushlow, C. A. , & Savage‐Dunn, C. (2018). BMP signaling determines body size via transcriptional regulation of collagen genes in *Caenorhabditis elegans* . Genetics, 210, 1355–1367. 10.1534/genetics.118.301631 30274988 PMC6283163

[acel13693-bib-0063] Madhu, B. , Lakdawala, M. F. , Issac, N. G. , & Gumienny, T. L. (2020). Caenorhabditis elegans saposin‐like spp‐9 is involved in specific innate immune responses. Genes Immun, 21, 301–310. 32770079 10.1038/s41435-020-0108-6 PMC7652716

[acel13693-bib-0034] McElwee, J. , Bubb, K. , & Thomas, J. H. (2003). Transcriptional outputs of the *Caenorhabditis elegans* forkhead protein DAF‐16. Aging Cell, 2, 111–121. 10.1046/j.1474-9728.2003.00043.x 12882324

[acel13693-bib-0035] Miller, H. A. , Dean, E. S. , Pletcher, S. D. , & Leiser, S. F. (2020). Cell non‐autonomous regulation of health and longevity. eLife, 9, e62659. 10.7554/eLife.62659 33300870 PMC7728442

[acel13693-bib-0036] Morimoto, R. I. (1998). Regulation of the heat shock transcriptional response: Cross talk between a family of heat shock factors, molecular chaperones, and negative regulators. Genes & Development, 12, 3788–3796. 9869631 10.1101/gad.12.24.3788

[acel13693-bib-0037] Morita, K. , Chow, K. L. , & Ueno, N. (1999). Regulation of body length and male tail ray pattern formation of *Caenorhabditis elegans* by a member of TGF‐beta family. Development, 126, 1337–1347. 10021351 10.1242/dev.126.6.1337

[acel13693-bib-0038] Morley, J. F. , & Morimoto, R. I. (2004). Regulation of longevity in *Caenorhabditis elegans* by heat shock factor and molecular chaperones. Molecular Biology of the Cell, 15, 657–664. 10.1091/mbc.E03-07-0532 14668486 PMC329286

[acel13693-bib-0039] Murphy, C. T. , McCarroll, S. A. , Bargmann, C. I. , Fraser, A. , Kamath, R. S. , Ahringer, J. , Li, H. , & Kenyon, C. (2003). Genes that act downstream of DAF‐16 to influence the lifespan of *Caenorhabditis elegans* . Nature, 424, 277–283. 10.1038/nature01789 12845331

[acel13693-bib-0040] Pfeffer, S. R. (2017). Rab GTPases: Master regulators that establish the secretory and endocytic pathways. Molecular Biology of the Cell, 28, 712–715. 10.1091/mbc.E16-10-0737 28292916 PMC5349778

[acel13693-bib-0041] Qi, W. , Yan, Y. , Pfeifer, D. , Donner, V. , Gromoff, E. , Wang, Y. , Maier, W. , & Baumeister, R. (2017). *C. elegans* DAF‐16/FOXO interacts with TGF‐ss/BMP signaling to induce germline tumor formation via mTORC1 activation. PLoS Genet, 13, e1006801. 10.1371/journal.pgen.1006801 28549065 PMC5467913

[acel13693-bib-0042] Ramakrishnan, K. , Ray, P. , & Okkema, P. G. (2014). CEH‐28 activates dbl‐1 expression and TGF‐beta signaling in the *C. elegans* M4 neuron. Developmental Biology, 390, 149–159. 10.1016/j.ydbio.2014.03.015 24690231 PMC4023489

[acel13693-bib-0043] Riedel, C. G. , Dowen, R. H. , Lourenco, G. F. , Kirienko, N. V. , Heimbucher, T. , West, J. A. , Bowman, S. K. , Kingston, R. E. , Dillin, A. , Asara, J. M. , & Ruvkun, G. (2013). DAF‐16 employs the chromatin remodeller SWI/SNF to promote stress resistance and longevity. Nature Cell Biology, 15, 491–501. 10.1038/ncb2720 23604319 PMC3748955

[acel13693-bib-0044] Roberts, A. F. , Gumienny, T. L. , Gleason, R. J. , Wang, H. , & Padgett, R. W. (2010). Regulation of genes affecting body size and innate immunity by the DBL‐1/BMP‐like pathway in *Caenorhabditis elegans* . BMC Developmental Biology, 10, 61. 10.1186/1471-213X-10-61 20529267 PMC2894779

[acel13693-bib-0045] Savage, C. , das, P. , Finelli, A. L. , Townsend, S. R. , Sun, C. Y. , Baird, S. E. , & Padgett, R. W. (1996). *Caenorhabditis elegans* genes sma‐2, sma‐3, and sma‐4 define a conserved family of transforming growth factor beta pathway components. Proceedings of the National Academy of Sciences of the United States of America, 93, 790–794. 10.1073/pnas.93.2.790 8570636 PMC40134

[acel13693-bib-0046] Scherz‐Shouval, R. , Santagata, S. , Mendillo, M. L. , Sholl, L. M. , Ben‐Aharon, I. , Beck, A. H. , Dias‐Santagata, D. , Koeva, M. , Stemmer, S. M. , Whitesell, L. , & Lindquist, S. (2014). The reprogramming of tumor stroma by HSF1 is a potent enabler of malignancy. Cell, 158, 564–578. 10.1016/j.cell.2014.05.045 25083868 PMC4249939

[acel13693-bib-0061] Schultz, R. D. , Bennett, E. E. , Ellis, E. A. , & Gumienny, T. L. (2014). Regulation of extracellular matrix organization by BMP signaling in caenorhabditis elegans. PLoS One, 9(7), e101929. 25013968 10.1371/journal.pone.0101929 PMC4094471

[acel13693-bib-0047] Stenmark, H. (2009). Rab GTPases as coordinators of vesicle traffic. Nature Reviews. Molecular Cell Biology, 10, 513–525. 10.1038/nrm2728 19603039

[acel13693-bib-0048] Suzuki, Y. , Yandell, M. D. , Roy, P. J. , Krishna, S. , Savage‐Dunn, C. , Ross, R. M. , Padgett, R. W. , & Wood, W. B. (1999). A BMP homolog acts as a dose‐dependent regulator of body size and male tail patterning in *Caenorhabditis elegans* . Development, 126, 241–250. 9847238 10.1242/dev.126.2.241

[acel13693-bib-0049] Taneja‐Bageshwar, S. , & Gumienny, T. L. (2013). Regulation of TGFbeta superfamily signaling by two separable domains of glypican LON‐2 in *C. elegans* . Worm, 2, e23843. 10.4161/worm.23843 24778932 PMC3875644

[acel13693-bib-0050] Taylor, R. C. , & Dillin, A. (2013). XBP‐1 is a cell‐nonautonomous regulator of stress resistance and longevity. Cell, 153, 1435–1447. 10.1016/j.cell.2013.05.042 23791175 PMC4771415

[acel13693-bib-0051] Tian, C. , Sen, D. , Shi, H. , Foehr, M. L. , Plavskin, Y. , Vatamaniuk, O. K. , & Liu, J. (2010). The RGM protein DRAG‐1 positively regulates a BMP‐like signaling pathway in *Caenorhabditis elegans* . Development, 137, 2375–2384. 10.1242/dev.051615 20534671 PMC2889605

[acel13693-bib-0052] Uno, M. , Tani, Y. , Nono, M. , Okabe, E. , Kishimoto, S. , Takahashi, C. , Abe, R. , Kurihara, T. , & Nishida, E. (2021). Neuronal DAF‐16‐to‐intestinal DAF‐16 communication underlies organismal lifespan extension in *C. elegans* . iScience, 24, 102706. 10.1016/j.isci.2021.102706 34235410 PMC8246587

[acel13693-bib-0053] Van Gilst, M. R. , Hadjivassiliou, H. , Jolly, A. , & Yamamoto, K. R. (2005). Nuclear hormone receptor NHR‐49 controls fat consumption and fatty acid composition in *C. elegans* . PLoS Biology, 3, e53. 10.1371/journal.pbio.0030053 15719061 PMC547972

[acel13693-bib-0054] Van Gilst, M. R. , Hadjivassiliou, H. , & Yamamoto, K. R. (2005). A *Caenorhabditis elegans* nutrient response system partially dependent on nuclear receptor NHR‐49. Proceedings of the National Academy of Sciences of the United States of America, 102, 13496–13501. 10.1073/pnas.0506234102 16157872 PMC1201344

[acel13693-bib-0055] Wang, J. , Tokarz, R. , & Savage‐Dunn, C. (2002). The expression of TGFbeta signal transducers in the hypodermis regulates body size in *C. elegans* . Development, 129, 4989–4998. 12397107 10.1242/dev.129.21.4989

[acel13693-bib-0056] Wang, R. N. , Green, J. , Wang, Z. , Deng, Y. , Qiao, M. , Peabody, M. , Zhang, Q. , Ye, J. , Yan, Z. , Denduluri, S. , Idowu, O. , Li, M. , Shen, C. , Hu, A. , Haydon, R. C. , Kang, R. , Mok, J. , Lee, M. J. , Luu, H. L. , & Shi, L. L. (2014). Bone Morphogenetic Protein (BMP) signaling in development and human diseases. Genes & Diseases, 1, 87–105. 10.1016/j.gendis.2014.07.005 25401122 PMC4232216

[acel13693-bib-0057] Watterson, A. , Tatge, L. , Wajahat, N. , Arneaud, S. L. B. , Solano Fonseca, R. , Beheshti, S. T. , Metang, P. , Mihelakis, M. , Zuurbier, K. R. , Corley, C. D. , Dehghan, I. , McDonald, J. G. , & Douglas, P. M. (2022). Intracellular lipid surveillance by small G protein geranylgeranylation. Nature, 605, 736–740. 10.1038/s41586-022-04729-7 35585236 PMC9885440

[acel13693-bib-0058] Welz, T. , Wellbourne‐Wood, J. , & Kerkhoff, E. (2014). Orchestration of cell surface proteins by Rab11. Trends in Cell Biology, 24, 407–415. 10.1016/j.tcb.2014.02.004 24675420

[acel13693-bib-0059] Zhang, X. , & Zhang, Y. (2012). DBL‐1, a TGF‐beta, is essential for *Caenorhabditis elegans* aversive olfactory learning. Proceedings of the National Academy of Sciences of the United States of America, 109, 17081–17086. 10.1073/pnas.1205982109 23019581 PMC3479501

[acel13693-bib-0060] Zugasti, O. , & Ewbank, J. J. (2009). Neuroimmune regulation of antimicrobial peptide expression by a noncanonical TGF‐beta signaling pathway in *Caenorhabditis elegans* epidermis. Nature Immunology, 10, 249–256. 10.1038/ni.1700 19198592

